# Construction and detection of the tissue-specific pINV-HPV16 E6/7 vector

**DOI:** 10.3892/ol.2014.2736

**Published:** 2014-11-25

**Authors:** HUI GAO, ZHENGFANG HUANG, CHENLONG SHI, HOUDA LI

**Affiliations:** 1 Department of Dermatology, Clinical Medical College, Yangzhou University, Yangzhou, Jiangsu 225001, P.R. China; 2 College of Veterinary Medicine, Yangzhou University, Yangzhou, Jiangsu 225000, P.R. China

**Keywords:** human papilloma viruses, E6/7, pINV-HPV16 E6/7, tissue-specificity

## Abstract

A tissue-specific promoter can control downstream gene expression in tissues or organs. The human involucrin (hINV) promoter (pINV) that contains 2474 bp of hINV upstream sequence is able to regulate tissue-specific gene expression. This tissue specificity may be important for the prevention and treatment of human papilloma virus infections. pINV was cloned by polymerase chain reaction and the human papillomavirus (HPV)16 E6/7 gene was obtained from the cancer tissue samples of patients with cervical carcinoma at the Yangzhou Maternal and China Health-Care Center of Jinagsu Province (Yangzhou, China). First, specific primers were designed according to the genomic DNA sequence of the HPV16-type standard strain that has been reported and the E6/7 gene was acquired by PCR. The carcinogenic fraction of the E6/7 gene was removed and the remaining section was cloned into T vectors, sequenced correctly and then cloned into the eukaryotic expression vector pCEP4, which was lacking the CMV promoter. The positive recombinants were identified using blue-white screening and endonuclease digestion, subsequent to sequencing and analysis, and the tissue-specific recombinant pINV-HPV16E6/7 plasmids was detected.

## Introduction

Cervical cancer is the second most common type of cancer in females worldwide, particularly in numerous developing countries (1). To reduce the high morbidity rate, the development of effective prevention and gene therapy is needed. With the advancement of molecular biology, gene therapy can be developed for a variety of cancers (1,2). Human papillomaviruses (HPVs) are double-stranded DNA viruses and, at present, >200 genotypes have been identified (3,4). Infection with high-risk human papillomaviruses is known to be the predominant risk factor for cervical cancer. HPVs can be detected in >99% of cervical cancer patients (5). HPVs encode two proteins, E6 and E7, which are important for cell immortalization. E6 and E7 interact with p53 and pRb, respectively (6,7), and induce growth inhibition via apoptosis (8). E7 leads to the functional inactivation of these proliferation regulators and uncontrolled cell proliferation (9). A previous study has revealed that the expression of HPV16 E6 may play an important role in cell transformation and cancer development (10). The tissue-specific promoter, also termed the organ specific promoter, can control the expression of downstream genes in tissues or organs and is closely associated with the serotonin receptor gene, similar to tissue-specific alternative promoters (11). Human involucrin (hINV) is selectively expressed in the stratified squamous epithelium, where it is associated with membrane protein (12,13). The region that can regulate tissue-specific expression is the hINVpromoter (pINV), which contains 2474 bp of hINV upstream sequence (14). pINV was cloned using polymerase chain reaction (PCR) and the HPV16 E6/7 was extracted from cervical cancer tissues obtained from patients at the Yangzhou Maternal and China Health-Care Center of Jinagsu Province (Yangzhou, China). First, the carcinogenic fraction was removed from the E6/7 gene and the remaining section was cloned into T vectors, correctly sequenced and then cloned into the eukaryotic expression vector pCEP4, which was separated from the CMV promoter. The tissue specificity of the recombinant pINV-HPV16E6/7 plasmid was detected in the present study.

## Materials and methods

### Materials

Surgical excisions of cervical cancer tissue samples were obtained from 13 patients (age range, 41–58 years) at the Yangzhou Maternal and China Health-Care Center of Jiangsu Province. The pathological diagnoses of these specimens were cervical squamous-cell carcinoma; 10 of which were stage II and three of which were stage III. The study was approved by the ethics committee of Yangzhou University (Yangzhou, China) and written informed consent was obtained from all participants. The pMD18-T vector, DNA Ligation kit and Recmobinant DNase I, RNase-free were purchased from Takara Biotechnology (Dalian) Co., Ltd. (Dalian, China). The expression vector pCEP4 was kindly provided by Professor Huaichang Sun, College of Veterinary Medicine, Yangzhou University and the host bacterium DH5α was obtained from the Comparative Medicine Center (College of Veterinary Medicine, Yangzhou University). DNA polymerase, the *Not*I, *Xho*I, *Hind*III and *Bam*HI restriction enzymes and DEPC were the products of Shanghai Sangon Biological Engineering Technology & Services Co., Ltd (Shanghai, China). QIAquick Gel Extraction kits were obtained from Qiagen (Hilden, Germany). The DNAExtraction Kit Ver.3.0 [Takara Biotechnology (Dalian) Co., Ltd.] and cell transfection kit (Fugene 6 transfection reagent) were purchased from Roche (Basel, Switzerland). The Eastep Universal RNA Extraction Kit was purchased from Promega (Madison, WI, USA). The yeast extracts and peptones for the bacterial culture were purchased from Oxoid (Basingstoke, Hampshire, UK). The reagents for microinjection, including M16, pregnant mare serum and human chorionic gonadotropin and the hyaluronic acid enzymes were purchased from Sigma-Aldrich (St. Louis, MO, USA). All other reagents were analytical reagents made in China.

### Cloning and sequence determination of the tissue-specific promoter gene, pINV, and HPV16 E6/7

The reference pINV gene primers were as follows: Upstream primer 1, 5′-TTGCGGCCG CAAGCTTCTCCATGTGTCATGT-3′; and downstream primer 2, 5′-TACTCGAGGAGCTGAGCAGGAGTCAGG-3′. The designed *Not*I restriction site was located at the 5′ end of the upstream primer and the designed *Xho*I restriction site was located at the 5′ end of the downstream primer. The E6/7 gene was designed with reference to the entire genome sequence of HPV16: Upstream primer 3, 5′-TGCTCGAGATGCACCAAAAGAGAACTG-3; and downstream primer 4, 5′-ATGGATCCTTATGGTTTCTGAGAA CAGATG-3′. The designed *Xho*I restriction sites were located in the 5′ end of the upstream primer and the designed *Bam*HI restriction sites were located in the 5′ end of the downstream primer. The tissue specimens from cervical cancer resection were obtained and the tissue DNA was extracted as formwork according to the manufacturer’s instructions for the DNA extraction kit. PCR was used for the amplification of the pINV gene and HPV 16E6/7 gene fragments and the PCR products were recovered using a gel extraction kit subsequent to 0.8% agarose gel electrophoresis. According to conventional methods, the fragments were ligated and transformed with the pMD18-T and pGEM-T Easy vectors. The positive colonies were then selected by blue-white selection and the positive colonies were added t 5 ml of Luria broth containing 100 μg/ml ampicillin culture medium at 37°C. The bacteria were cultivated for ~12 h and the alkaline lysis method for extraction of plasmid DNA was then utilized. The recombinant T-pINV plasmids were appraised by double restriction enzyme digestion using the *Not*I and *Xho*I restriction enzymes, and the recombinant pGEM-T-HPV16 E6/7 was assessed by double restriction enzyme digestion using the *Xho*I and *Bam*HI restriction enzymes. Takara Biotechnology Company sequenced the pINV and HPV16E/7 gene and completed the HPV16 E6/7 gene transformation, where the E6 gene of leucine codon 50 (TTA), is mutated to glycine (GGT) and the E7 gene 24 and 26 amino acid codons are modified to glycine (GGT and GGG). The sequencing of the pINV and HPV16E/7 genes was analyzed using DNA Star software (DNA Star Inc., Madison, WI, USA).

### Transformation of the pCEP4 vector into cells and construction of the recombinant pCEP4/pINV(-PCMV) plasmid

**Figure 1:**
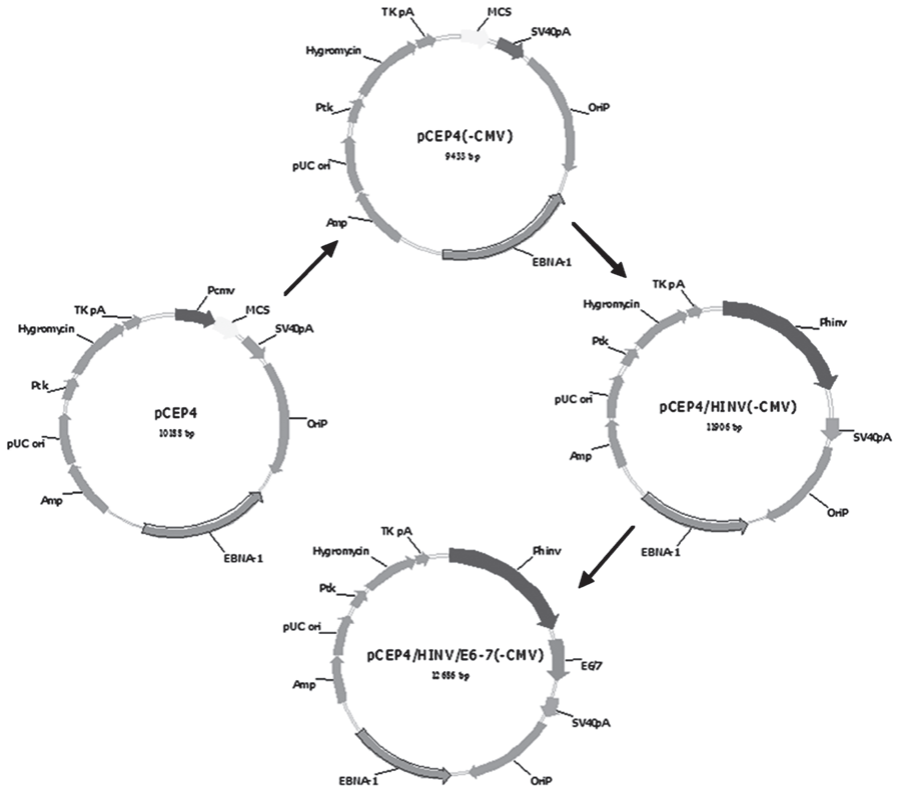
Entire process of the construction of the recombinant pINV-HPV16 E6/7 plasmid. pINV, human involucrin promoter; HPV, human papillomavirus.

The PCMV plasmid was digested by *Bgl*II to remove the original promoter and the digested PCMV then self-linked to give the pCEP4(-PCMV) plasmid. The pCEP4(-PCMV) and PUC-18 T-pINV plasmids were digested by *Not*I and *Xho*I, respectively, and the pCEP4(-PCMV) vector and the prime pINV gene were recovered using a gel extraction kit (Horan Bio Technologies Co., Ltd., Shanghai, China). The recycling products were mixed in a 3:1 molar ratio, the same volume of solution I was added and then the cells were incubated at 4°C overnight. The cells transformed with the recombinant plasmid were plated and the positive colonies were selected to obtain the required pCEP4/pINV(-PCMV) plasmid. The recombinant pINV-HPV16E6/7 plasmid was then constructed. The pCEP4(-PCMV) and pGEM-T-HPV16 E6/7 plasmids were digested by *Bam*HI and *Xho*I, respectively, mixed with the E6/7 gene and the pCEP4/pINV(-PCMV) vector in a 3:1 ratio, the same volume of the solution I was added and the cells were incubated at 4°C overnight. The cells transformed with the recombinant plasmid were plated and the positive colonies were selected to obtain the required pINV-HPV16E6/7 plasmid. A schematic diagram of the entire process is shown in Fig. 1.

### Identification of the tissue-specific pINV

The manufacturer’s instructions for the Fugene 6 transfection reagent were followed in order to transfect the plasmid into the brain glioma, SP2/0, L929 and HeLa cells (all obtained from Shanghai Institutes for Biological Sciences of Chinese Academy of Sciences, Shanghai, China). The primers were designed according to the sequence of the recombinant pINV-HPV16E6/7 plasmid. The sequences of the upstream forward and the downstream reverse primers were 5′-CACAGGAGCGACCCAGAAAGTTA-3′ and 5′-GCTGGG TTTCTCTACGTGTTCTT-3′, respectively. It was estimated that the amplified DNA was 438 bp in length. The mRNA that was transfected into the brain glioma, SP2/0, L929 and HeLa cells was extracted according to the manufacturer’s instructions for the TRIzol Plus Purification kit (Invitrogen Life Technologies, Carlsbad, CA, USA). cDNA was amplified using the Super Script kit (Invitrogen Life Technologies) according to the manufacturer’s instructions. A total of 10 μl cDNA was mixed with 4 μl 10X PCR buffer, 2 μl (25 mM) MgCl_2_, 0.5 μl (100 pmol) of each primer, 32.7 μl distilled water and 0.3 μl (2.5 U) Taq DNA polymerase. The PCR reaction conditions were as follows: 35 cycles of 30 sec at 94°C, 30 sec at 50°C and 2 min at 72°C. The amplified products were sequenced to confirm pINV-HPV16E6/7 DNA (Shanghai Sangon Biological Engineering Technology & Services Co., Ltd.).

## Results

### Gene cloning and identification of the tissue-specific pINV

**Figure 2:**
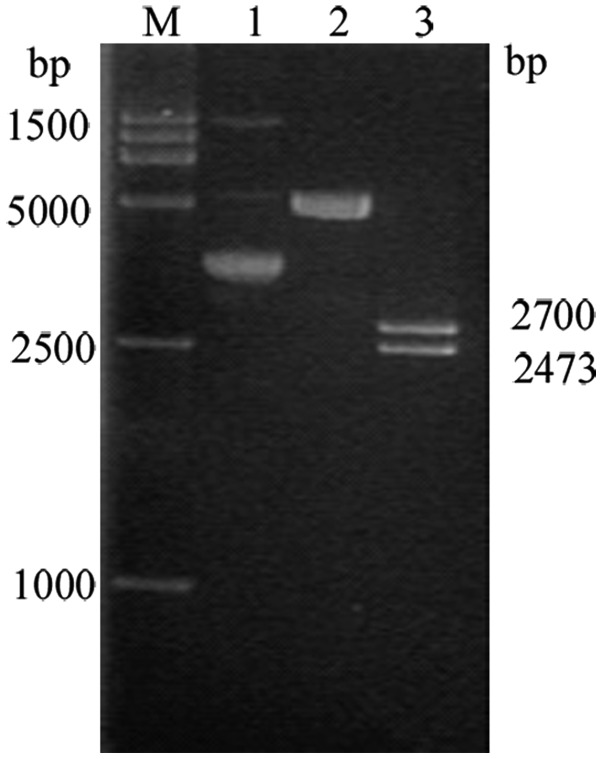
Identification of pMD18-T/pINV. Lane M, marker; lane 1, pMD18-T-pINV plasmid; lane 2, digestion by *Xho* yielded a fragment 5,200 bp in length; lane 3, double digestion by *Not* and *Xho* yielded fragments 2,692 and 2,474 bp in length. pINV, human involucrin promoter.

pINV fragments were amplified using PCR. A distinct band at 2,474 bp could be observed on 1% agarose gel electrophoresis. The fragments were recovered and ligated with PGEM-T. Cells were transformed with the resulting plasmid and the cells positive for the pMD18-T-pINV plasmid were selected using the blue-white screening method. Bands containing fragments 2474 and 2692 bp in size were obtained during double enzyme digestion using *Not*I and *Xho*I (Fig. 2).

### Cloning and identification of the pINV-HPV16E6/7 plasmid

**Figure 3:**
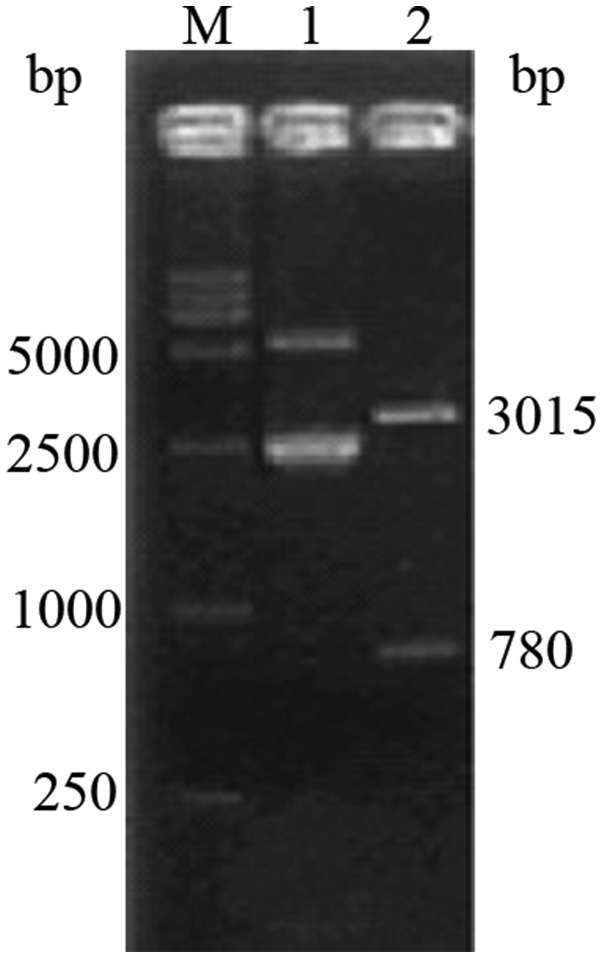
Identification of T Easy/E6/7. Lane M, marker; lane 1, pGEM-T-E6/7 plasmid; lane 2, double digestion by *BamH* and *Xho* yielded fragments 3,015 and 780 bp in length. pINV, human involucrin promoter.

The E6/7 fragment was amplified using PCR and 1% agarose gel electrophoresis revealed a clear band at 780 bp. The fragments were recovered and ligated using pGEM-T Easy vector (Promega, Madison, WI, USA), introduced to cells and the cells positive for the pGEM-T-E6/7 plasmid were selected using the blue-white method. Double enzyme digestion by *Bam*HI and *Xho*I yielded visible bands at 3,015 and 780 bp on 1% agarose gel electrophoresis (Fig 3).

### Replacement of the promoter within the vector with pINV

**Figure 4:**
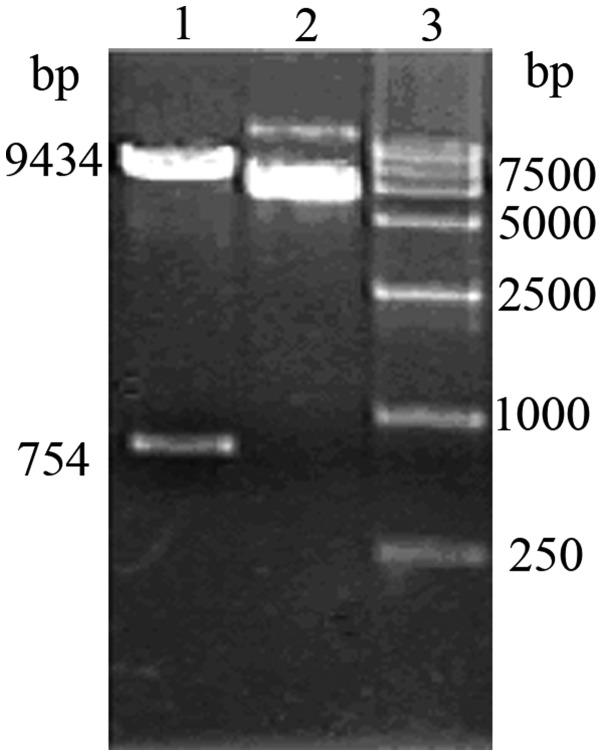
Identification of the pCEP4 plasmid. Lane 1, digestion by *Bgl* yielded fragments 9435 and 753 bp in length; lane 2, pCEP4 plasmid; lane 3, DL15000 marker.

**Figure 5:**
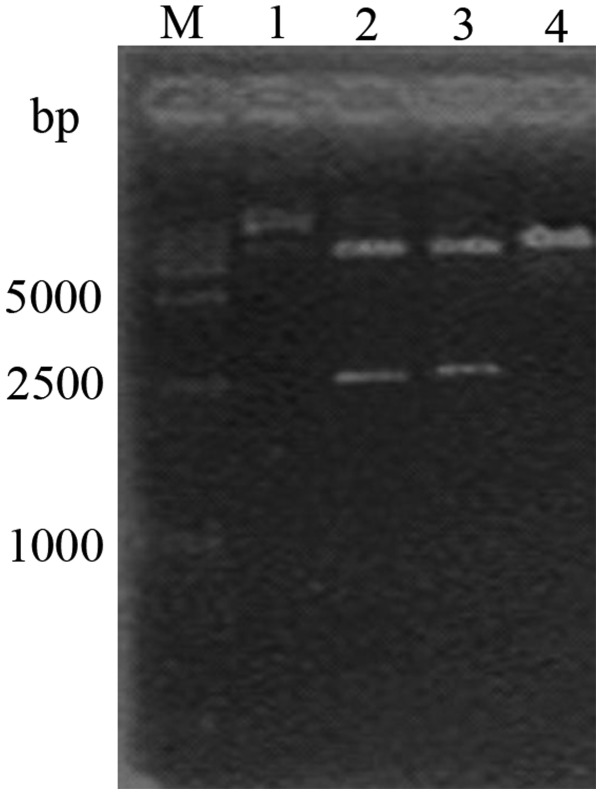
Identification of the recombinant pCEP4/pINV(-PCMV) plasmid. Lane M, DL15000 marker; lane 1, digestion by *Bgl*; lane 2, double digestion by *Not* and *Xho*; lane 3, double digestion by *Not* and *BamH*; lane 4, pCEP4/pINV(-PCMV) plasmid. pINV, human involucrin promoter.

Following the removal of the original promoter of the pCEP4 vector by enzyme digestion using *Bgl*II, 1% agarose gel electrophoresis revealed clear bands at 753 and 9,435 bp (Fig. 4) The fragments obtained by double digestion of the pCEP4(-PCMV) plasmid using *Not*I and *Xho*I were recovered and linked with the recovered fragments from pINV. The resulting plasmid was then introduced into cells and the cells positive for the recombinant pCEP4/pINV(-PCMV) plasmid were selected. The cells were identified as positive for the plasmid by single digestion using *Bgl*II, yielding a fragment of 11.9 kb and double digestion using *Not*I and *Xho*I, and *Not*I and *Bam*HI, respectively, yielding fragments of 2,474 bp and 9,435 bp in size, respectively, on 1% agarose gel electrophoresis, which was consistent with the expected fragment size (Fig. 5).

### Construction and identification of the recombinant pINV-HPV16E6/7 plasmid

**Figure 6:**
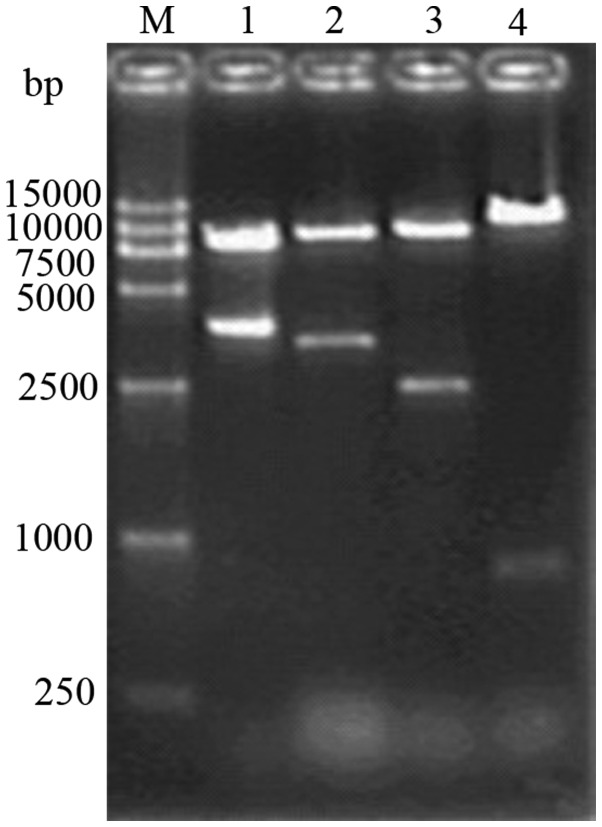
Identification of the pINV/HPV16E6/7 recombinant plasmid. Lane M, DL15000 marker; lane 1, digestion by *Sal* yielded fragments 8.9 and 3.8 kb in length; lane 2, double digestion by *Not* and *BamH* yielded fragments 9.5 and 3.2 kb in length; lane 3, double digestion of *Not* and *Xho* yielded fragments 10.2 and 2.5 kb in length; lane 4, double digestion of *Xho* and *BamH* yielded fragments 11.9 and 780bp in length. pINV, human involucrin promoter; HPV, human papillomavirus.

Following the double digestion of the pGEM-T-E6/7 plasmid, the E6/7 fragments were recovered and linked with the recovered pCEP4/pINV(-PCMV). The product was then introduced into cells and the cells positive for the plasmids were selected. The plasmids were identified by single and double digestion using *Sal*I, *Not*I and *Bam*HI, and *Not*I and *Xho*I, respectively. On 1% agarose gel electrophoresis, the *Xho*I and *Bam*HI double digestion yielded visible bands at 8.9, 3.8, 9.5, 3.2, 10.2, 2.5 and 11.9 kb, and 780 bp (Fig 6), which were consistent with the expected fragment sizes.

### Identification of the tissue-specific pINV

**Figure 7:**
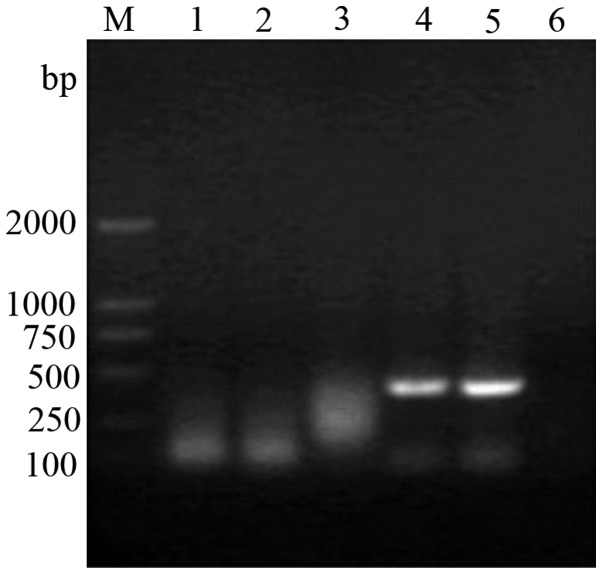
HPV16E6 mRNA expression of various cells. Lane M, DL15000 marker; lane 1, glioma cells; lane 2, L929 cells; lane 3, SP2/0 cells; lane 4, HeLa cells; lane 5, positive control; lane 6, blank control.

The recombinant pINV-HPV16E6/7 plasmid was transfected into cells derived from various tissues. RT-PCR and 1% agarose gel electrophoresis identified that the HPV16E6 mRNA was only expressed in the transfected HeLa cells, but was not expressed in the other cells (Fig. 7).

## Discussion

pINV is a tissue-specific promoter that is ~2474 bp in length and possesses two important regulatory regions, a distal regulatory region (DRR) and a proximal regulatory region (PRR). There are five binding sites (from AP1-1 to AP1-5) in these regions, and the existence of these sites is closely associated with the activity of the promoter and the subsequent gene transcription levels (15). The DRR (-2474/-1953 bp) mainly regulates downstream gene expression in keratinocyte cells near the surface and the PRR (-986/-41 bp) mainly regulates downstream gene expression in the inner layer of keratinocyte cells. The -1953/-986 bp region is mainly associated with the different expression model of downstream genes (5–13). HPV is a type of DNA virus and its rate varies according to ethnicity (16). The virus is also tissue-specific and is associated with human skin and mucous membrane tumors. In recent years, sexually transmitted diseases and cervical cancer caused by HPV infection have exhibited an ascendant trend, and diseases associated with HPV infection are difficult to cure (17). Currently, gene therapy has become an important method for cancer treatment and targeted gene therapy is the hot spot, linking the therapeutic gene with a tissue-specific promoter (18). A previous study has revealed that tissue-specific promoters play an important role in gene therapy for prostate cancer (19), and the specific expression of the therapeutic gene in the target tissue can reduce the side effects and improve the treatment effects. Establishing a skin-specific promoter would have a role in the treatment of HPV infection diseases by directing the therapeutic gene to the target skin and mucous membrane. In the present study, the eukaryotic vector pCEP4 was used as a skeleton and the tissue-specific pINV was inserted following the removal of the CMV promoter. Subsequently, the HPV16 E6/7 gene was inserted downstream of the pINV, following the removal of the cancer-causing section, resulting in the pINV-HPV16E6/7 recombinant plasmid. The tissue-specificity of the pINV was judged through the detection of HPV16 E6/7 expression in various types of cells. The recombinant pINV-HPV16E6/7 plasmid was transfected into brain glioma, SP2/0, L929 and HeLa cells. RT-PCR and 1% agarose gel electrophoresis revealed that mRNA was only expressed in the transfected HeLa cells and that there was no expression in the other cells. In conclusion, pINV in the recombinant pINV-HPV16E6/7 plasmid is expressed in a skin-specific manner. At present, no effective method for the prevention and tratement of HPV infection has been identified. Thus, the development of an effective vaccine against HPV may lead to a decrease in the morbidity and mortality rates of cervical cancer. However, it is difficult to obtain a large number of HPV viral particles *in vitro* to produce the traditional dead or attenuated viral vaccines, which has subsequently limited the development of HPV vaccines. The E6 and 7 proteins are considered ideal target antigens for therapeutic HPV vaccines, however, they exhibit carcinogenicity. In the present study, the carcinogenic fraction of the PV16E6/7 gene was removed and a pINV-HPV16E6/7 recombinant plasmid with skin tissue specificity was constructed. Future *in vivo* studies, which investigate whether this recombinant plasmid induces humoral and cellular immune responses are required.
